# Combining Exercise and Carbohydrate Restriction in Patients with Type 2 Diabetes Mellitus—A Critical Look at Possible Intervention Effects

**DOI:** 10.3390/ijerph192316251

**Published:** 2022-12-05

**Authors:** Samir Akrimi, Christian Brinkmann

**Affiliations:** 1 Institute of Cardiovascular Research and Sport Medicine, German Sport University Cologne, 50933 Cologne, Germany; 2 Department of Fitness & Health, IST University of Applied Sciences Düsseldorf, 40233 Düsseldorf, Germany

**Keywords:** type 2 diabetes mellitus, exercise, physical activity, nutrition, low-carbohydrate diet, ketosis

## Abstract

Combining regular exercise and a healthy diet is recommended in international guidelines to fight type 2 diabetes mellitus (T2DM). Low- and very low-carbohydrate diets have attracted attention in the last years. This article takes a critical look at the possible effects when regular exercise and carbohydrate restriction are combined. An increased intervention effect on the oxidative capacity as well as glucose and lipid profiles can be assumed (at least for a short period of time). However, anabolic signaling pathways might be blunted during a very low-carbohydrate diet and increasing ketosis. Thus, muscle build-up can become difficult or impossible. Furthermore, maximal performance during high-intensity workouts may be attenuated due to a possible reduced anaerobic glycolysis and metabolic inflexibility in T2DM patients. However, more studies are needed to evaluate the effects of this combination in comparison to those of exercise and other types of diet.

## 1. Introduction

According to the International Diabetes Federations’ 10th Diabetes Atlas, about 537 million people between 20 and 79 years are living with diabetes. If the number rises as predicted, the number of people living with diabetes will increase to 783 million by 2045. Type 2 diabetes mellitus (T2DM) is the most prevailing type, with a proportion of 90% of all global diabetes cases [1]. Therefore, effective interventional strategies to fight T2DM are urgently needed. Lifestyle interventions combining physical activity programs and a healthy diet are recommended in diabetes guidelines [2].

While evidence accumulates that carbohydrate restriction can improve metabolic health in T2DM patients [3,4], one can assume that additive effects can arise when exercise and carbohydrate restriction are combined [5].

However, there are some critical issues that have to be considered regarding the maximization of intervention effects. There can be a reduction in the availability of insulin as an anabolic hormone and a reduced anabolic signaling in skeletal muscle [6]. This could be disadvantageous with regard to a planned muscle build-up [7]. Furthermore, carbohydrates are well used substrates in anaerobic energy metabolism [8]. It can be speculated that a strong restriction of carbohydrates may thus reduce physical performance during high-intensity training sessions and diminish training adaptations [9,10].

This opinion article will critically discuss the role of carbohydrate restriction in the context of an exercise-based intervention for individuals with T2DM, and it will open future research perspectives.

## 2. Definitions of a Carbohydrate Diet

It is not precisely defined what a carbohydrate diet characterizes. Some studies define carbohydrate diets as a certain percentage of total macronutrient consumption per day or a certain amount of daily carbohydrates [11]. Feinman et al. [12] suggest definitions for types of a carbohydrate diet. A very low-carbohydrate ketogenic diet (VLCKD) consists of less than 10% carbohydrates of the total energy/day or 20–50 g/day, whether ketosis takes place or not. A low-carbohydrate diet (LCD) comprises less than 26% of the total energy/day or less than 130 g/day. In general, it must also be considered that clinical studies investigating low- or very low-carbohydrate diets do not always offer information on the quality of the carbohydrate-containing foods (glycemic index/load, fiber content, vitamins, etc.). The different “low-carb” definitions make it difficult to compare study outcomes [13]. It should further be mentioned that many studies only refer to a low- or very low-carbohydrate intake without mentioning the increase of other dietary components. For the sake of completeness, the definition of a diet with carbohydrate restriction should always go hand-in-hand with a statement about the dietary factor(s) that should be increased, e.g., a higher fat or protein consumption [14].

## 3. Effects of Carbohydrate Restriction in Individuals with Type 2 Diabetes Mellitus

A recent meta-analysis performed by Rafiullah et al. [4] analyzed the impact of VLCKDs in T2DM patients. They compared this type of diet to any recommended diet for people with T2DM. After 3 and 6 months, the VLCKD was superior in reducing glycated hemoglobin (HbA1c) levels and body weight. After 12 months, the VLCKD was still superior in reducing triglyceride levels, increasing high-density lipoprotein (HDL) levels, and reducing the use of anti-diabetic medication, but not any longer in improving HbA1c or body weight.

Another meta-analysis performed by Huntriss et al. [3] compared randomized controlled trials (RCTs) of an LCD to usual care. The meta-analysis showed a significant advantage in the LCD groups in improving HbA1c, triglyceride and HDL levels, and systolic blood pressure after 1 year. No significant differences were found for weight loss, total cholesterol, and low-density lipoprotein (LDL) levels or diastolic blood pressure.

In conclusion, a reduction of carbohydrate intake seems promising in improving the metabolic health of T2DM patients. An LCD seems to be feasible, while adherence to VLCKDs may be much more challenging because patients have to avoid more carbohydrate-containing foods to meet the stricter limits.

## 4. Effects of Combining Carbohydrate Restriction and Exercise in Individuals with Type 2 Diabetes Mellitus

In addition to a healthy diet, exercise training is a cornerstone in managing type 2 diabetes and can prevent its onset and progression [2]. Exercise has acute effects on skeletal muscle glucose uptake [15]. Chronic exercise can result in statistically significant decreases in HbA1c levels, improvements in the lipid profile, and increases in cardiovascular health [16].

A meta-analysis by Liu et al. [17] investigated the impact of high-intensity interval training (HIIT) compared to moderate-intensity continuous training (MICT) on health variables in patients with type 2 diabetes. In comparison, HIIT resulted in a more effective increase in maximal oxygen uptake (VO_2peak_), and improvements in HbA1c levels and lipid profile (LDL cholesterol). HIIT can be an efficient alternative to MICT.

The question arises whether physical training (with moderate-intensity and/or high-intensity exercise) and carbohydrate restriction can be successfully combined.

While studies involving T2DM patients are still missing, Perissiou et al. [18] have demonstrated in a study involving obese individuals that an 8-week LCD combined with moderate- and high-intensity exercise (endurance and strength training) was superior compared to a mixed diet and regular exercise in terms of improvements in VO_2peak_ and fat mass index. Furthermore, they have also shown that achieving a ketogenic state was associated with greater improvements in fat loss and inflammation.

In general, it can be assumed that a diet with carbohydrate restriction leads to alterations in metabolic pathways. Following exercise, there can be an intensified activation of 5′adenosine monophosphate-activated protein kinase (AMPK) and peroxisome proliferator-activated receptor gamma coactivator 1-alpha (PGC1α), leading to an up-regulation of skeletal muscle oxidative capacity [6].

However, there could also be some unfavorable aspects. Perissiou et al. [18] found greater reductions in lean muscle mass in their study with obese patients. The authors attribute the greater reductions in lean muscle mass to an increased loss of water (which is part of the lean muscle mass) as ketosis is associated with increased water excretion [19]. During a strong carbohydrate restriction, a loss of muscle mass could also be attributable to increased proteolysis due to strongly reduced insulin levels in parallel to ketosis [20]. Furthermore, the insulin-like growth factor 1 (IGF-1)/AKT/mTOR pathway can be blunted. Subsequently, gaining muscle mass can become difficult or impossible, even despite energy sufficiency [6]. As muscle hypertrophy should be one of the main training goals in diabetes to increase glucose-consuming body mass, this can be seen very critically.

There could be another critical point for the combination of carbohydrate restriction and training outcomes. The carbohydrate need of skeletal muscle increases as exercise intensity increases [8]. If carbohydrate availability is limited, there will be a metabolic switch to fat and ketones as the primary fuel sources [21]. This may be advantageous for prolonged exercise since fat stores are of extensive quantity [21]. In contrast, for high-intensity exercise, this suggests that training effects could be mitigated due to limited performance during the training sessions. Should one, therefore, deduce that a diet with carbohydrate restriction would be better only combined with moderately intense exercise to maximize the training effects in patients with T2DM? Currently, no study involving T2DM patients exists to answer the question whether carbohydrate restriction and HIIT get along well. However, in this context, Cipryan et al.’s study [22] demonstrated that a 4-week adaptation period to a very low-carbohydrate high-fat diet preserved high-intensity exercise performance (compared to the performance of a group with a mixed Western-based diet) in a group of healthy individuals. It is questionable whether these results are transferable to T2DM patients, considering that patients with T2DM can show a certain degree of metabolic inflexibility [9]. Future studies are warranted.

Another idea would be to combine the approaches one after the other. In Asle Mohammadi Zadeh et al.’s study [23], the patients with T2DM performed an LCD for 8 weeks, then performed 12 weeks of HIIT followed by 4 further weeks of diet. Their results demonstrated that HIIT, along with a low-carbohydrate regime, improves cardiovascular variables and reduces pro-inflammatory and increases anti-inflammatory markers.

## 5. Conclusions

From our point of view, it seems likely that the combination of carbohydrate restriction and exercise can be effective in improving T2DM patients’ health. However, one should not get too enthusiastic early on, since studies have yet to demonstrate superior effects compared to exercise and other forms of diet (e.g., mixed or low-fat diet) in patients with T2DM.

In addition, there are some critical aspects. Particularly when a very low- carbohydrate diet is performed, the risk of increased loss of muscle mass and the difficulty of building muscles must be considered. Furthermore, there can be reductions in maximal anaerobic performance during high-intensity training programs. Individual responses should be taken into account.

We call for the undertaking of standardized studies that clearly define which type of diet in direct combination with which type of exercise program produces which effects in T2DM patients.

It must also be considered how exactly these diets are performed. The question remains which other macronutrients are increased and to what extent (e.g., low-carbohydrate high-fat, or low-carbohydrate high-protein diet). LCDs could tend to result in reduced intake of fiber and fruits as well as increased intake of “bad” lipids (e.g., trans fats), all of which are risk factors for cardiovascular diseases and premature death [24]. In this context, it should be pointed out that there are studies that indicate an increased mortality rate with long-term carbohydrate restriction [24,25]. Even if beneficial short-term effects of a diet with carbohydrate restriction (for up to 1 year) have been reported in T2DM, long-term effects (>1 year) need to be further investigated. Healthy food choices should be practiced and encouraged.

As safe advice for patients, we recommend trying the combination of a short-term LCD and exercise, with regular metabolic health and fitness checks at the patients’ physician.

## Data Availability

Not applicable.
